# 电磁导航支气管镜引导下注入荧光剂在肺结节定位切除术中可行性

**DOI:** 10.3779/j.issn.1009-3419.2020.103.01

**Published:** 2020-06-20

**Authors:** 功铭 王, 勇斌 林, 孔嘉 罗, 晓丹 林, 兰军 张

**Affiliations:** 510060 广州，中山大学肿瘤防治中心胸外科 Department of Thoracic Surgery of Sun Yat-sen University Cancer Center, Guangzhou 510060, China

**Keywords:** 肺结节, 磁导航支气管镜, 吲哚菁绿, 荧光胸腔镜, Pulmonary nodule, Magnetic navigation bronchoscope, Indocyanine green, Fluorescence thoracoscope

## Abstract

**背景与目的:**

临床上肺内体积小，位置深且无实性成分的结节在电视辅助胸腔镜手术下切除给外科医生带来很多挑战。本研究的目前在于探讨经磁导航支气管镜（electromagnetic navigation bronchoscope, ENB）引导下肺部结节注入吲哚菁绿（indocyanine green, ICG）定位及在荧光腔镜下肺部小结节切除的可行性。

**方法:**

2018年12月-2019年8月，我院连续16例共计30个肺周围病变患者接受了荧光胸腔镜下肺部结节切除术。术前均行磁导航引导注入ICG定位。

**结果:**

所有患者均先行磁导航引导下肺结节定位术，染色完成后立即行手术切除。结节的平均大小为（11.12±3.65）mm。平均导航时间为（12.06±2.74）min，染料标记与肺部手术切除的平均间隔为（25.00±5.29）min。所有病灶均被完全切除，定位成功率100.00%，无出血及其他并发症发生，术后病理结果证实定位的准确性。

**结论:**

经磁导航引导下注入荧光染剂是一种新颖且有效的方法，可以定位肺部微小不可触及的病灶。这种方法可帮助外科医生更快更方便的识别病灶，实用性强，值得推广。

## Abstract (en)

**Background and Objective:**

It is a great challenge for surgeons to resect pulmonary nodules with small volume, deep position and no solid components under video-assisted thoracoscopic surgery. The purpose of this study is to explore the feasibility and necessity of the localization of pulmonary nodules by injecting indocyanine green (ICG) under the guidance of magnetic navigation bronchoscope and the resection of small pulmonary nodules under the fluoroscope.

**Methods:**

Between December 2018 and August 2019, sixteen consecutive patients with 30 peripheral lung lesions in our hospital received fluorescent thoracoscopic pulmonary nodule resection. Electromagnetic navigation bronchoscope (ENB) was performed before surgery to guide ICG to the target lesion.

**Results:**

All patients underwent magnetic navigation-guided pulmonary nodule localization, and surgical resection was performed immediately after localization was completed. The average size of the nodules was (11.12±3.65) mm. The average navigation time was (12.06±2.74) minutes, and the average interval between dye labeling and lung resection was (25.00±5.29) minutes. All lesions were completely resected, the localization success rate was 100.00%, no bleeding and other complications occurred after the localization, the postoperative pathological results confirmed the accuracy of the staining.

**Conclusion:**

Indocyanine green injection under the guidance of magnetic navigation bronchoscope is an effective way to locate pulmonary nodules, which can locate small and untouchable lesions in the lung. This method can help surgeons identify lesions more quickly and accurately. It is practical and worthy of promotion.

目前肺癌是世界上发病率和死亡率最高的恶性肿瘤^[1]^。根据2015年中国癌症统计数据，中国肺癌的发病率和死亡率排名第一^[2]^。大多数原因是一旦被诊断出肺癌患者已处于晚期，并且治疗效果差，5年生存率仅为15%-16%^[3]^。近年来，随着低剂量电子计算机断层扫描（low-dose computer tomography, LDCT）在肺癌早期筛查中的应用，肺癌的早期检出率提高了12%，病死率降低了20%^[4]^。但是其中大多数结节很难从影像上确定其良恶性。临床上如患者肺部结节具有毛刺征、血管征、支气管征、胸膜牵拉等这些高危影像特征，均提示恶变可能^[5]^。对于磨玻璃结节（ground glass nodules, GGN）这样的病变，很少有实性成分，术者难以通过传统的手指触摸或者器械辅助来识别结节的位置，这给外科医生尤其是年轻医生带来了巨大挑战，有报道传统方法定位失败进而中转开胸的比例高达46%^[6]^。既往有很多研究报道了术前用于肺结节定位的方法，例如电子计算机断层扫描（computed tomography, CT）引导下带钩钢丝定位、弹簧圈定位或注入染料定位、放射性核素定位等方法。然而CT引导下这些定位方法需要患者定位后限制活动，否则容易脱落而造成手术失败，再者易引起气胸，穿刺部位疼痛及肺实质出血等并发症。此外患者如果长期吸烟或从事粉尘相关工作，肺表面呈碳沉着样改变，难以辨别染料具体位置，可能导致切缘不够或者未切到等手术风险^[7]^。电磁导航支气管镜技术（electromagnetic navigation bronchoscope, ENB）是将磁导航技术和支气管镜检查术以及三维重建技术相整合起来的新手段。该技术原理是利用体外磁场定位板来引导气道内探头来进行靶病灶的定位及活检，既往有证实了ENB术中用于定位的高成功率，Awais、Brown、Luo等^[8, 9, 15]^的研究均证实ENB用于肺结节定位且均成功切除，不需要额外开胸手术。本研究的目的是评估使用ENB引导注入ICG定位加荧光腔镜下肺切除术来治疗肺部微小且难以触及病变的可行性。

## 1 资料与方法

### 1.1 临床资料

#### 1.1.1

我们回顾分析了2018年12月-2019年8月期间于中山大学肿瘤防治中心胸外科行术前ENB荧光染料吲哚菁绿定位加术后行荧光腔镜下肺切除的患者。最终入组16例患者包含30个结节，其中单发病灶患者9例，双发病灶患者2例，3处病灶患者3例，4处病灶患者2例，患者一般情况见表 1。这项研究得到中山大学癌症中心伦理委员会的批准，术前所有患者均已获得知情同意。

**1:** 本研究中16例患者的临床特征 Clinical characteristics of 16 patients

Variables	Data
Gender	
Male	7 (43.8%)
Female	9 (56.2%)
Age (Mean±SD, yr)	56.19±12.74
Past history	
Yes	5 (31.3%)
No	11 (68.7%)
Family history	
Yes	10 (62.5%)
No	6 (37.5%)
Smoking history	
Yes	4 (25.0%)
No	12 (75.0%)
Symptoms	
Yes	5 (31.3%)
No	11 (68.7%）
Tumor marker	
Normal	10 (62.5%)
Unnormal	6 (37.5%）
Number of lesions	
1	9 (56.3%)
2	2 (12.5%
3	3 (18.7%)
4	2 (12.5%)

#### 1.1.2 入选标准

① 年龄在18岁-85岁之间；②临床怀疑为恶性，且无全身转移；③肺结节直径≤20mm，位于周围实质中；④病灶可切除且无手术禁忌证。

#### 1.1.3 排除标准

① 年龄大于85岁或小于18岁者；②有全身转移；③结节 > 20mm；④一般情况差，患者心肺功能难以耐受手术切除；⑤签署同意书，拒绝手术者。

#### 1.1.4 设备及手术方式

使用SuperDimension（美敦力，上海，中国）软件，患者术前均完善1mm胸部薄层CT增强与平扫检查。结果导入软件，使用患者CT扫描像的轴向，冠状和矢状面视图来构建3D气道树，构建通向肺结节的线路图，此外对于行肺段切除患者术前均行3D重建明确有无血管及气管变异。

#### 1.1.5 手术过程

患者先取平卧位，使用单腔管气管吸入性麻醉，麻醉完成后实行手术，根据术前重建好的线路图，借助导航控制台、可定位的电磁导管和延长的工作导管定位每个结节。在导航过程中，可以测量出可定位的电磁导管与结节之间的距离，病变定位后，将可带有延长导管的定位电磁导管的位置调整到距最近胸膜表面1.5cm以上，以避免穿孔，并将可定位的电磁导管从延长工作导管中退出。然后将吲哚菁绿染料0.1mL通过延长管注入结节中间部位，注射完成后退出延长管，使用气管镜清理冲洗气道分泌物，观察有无出血气道损伤等并发症，见图 1A-图 1F。

**1:**
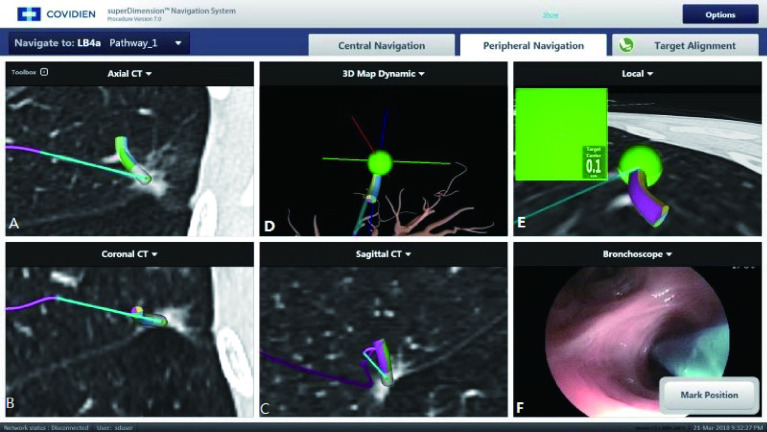
电磁导航支气管镜引导下定位过程。根据术前建立的线路图将传感器探头送至距靶病变1.0 cm处，A、B、C分别显示探头在冠状位及矢状位下探头与肺結节关系模式图；D、E表示3D重建视图下肺探头与結节位置图; F表示将荧光染料0.1 mL ICG注入到结节处，并反复来回抽插导丝，使結节充分染色，定位完成后清理冲洗气道分泌物，观察有无出血及起气道损伤。 Electromagnetic navigation bronchoscope positioning process. According to the map established before surgery, send the sensor probe to 1.0 cm away from the target lesion. A, B, and C respectively show the relationship between the probe and the lung nodules in the coronary and sagittal positions; D and E show the location of the probe and the nodule during positioning; F shows the injection of fluorescent dye 0.1 mL ICG into the nodule. After the positioning is completed, the airway secretions are cleaned up, and the postoperative bleeding and airway damage are observed.

在定位和荧光剂标记完成后，改用双腔气管导管，患者取侧卧位，所有病例均采用两切口即上肺取腋中线的第七肋间，中、下肺取第八肋间作为腔镜镜孔；下肺取腋前线第四肋间，中、下肺取第五肋间4cm作为操作孔，逐层开胸进胸腔，如图 2A，结节在荧光腔镜下呈绿色，使用美敦力器械肺组织楔形切除送冰冻，如果诊断为恶性（不包括非典型腺瘤性增生，原位癌，微小浸润癌），术前检查心肺功能耐受手术切除则行标准肺叶切除加纵膈淋巴结清扫，否则只行亚肺叶切除加纵隔淋巴结活检术，见图 2A-图 2F。

**2:**
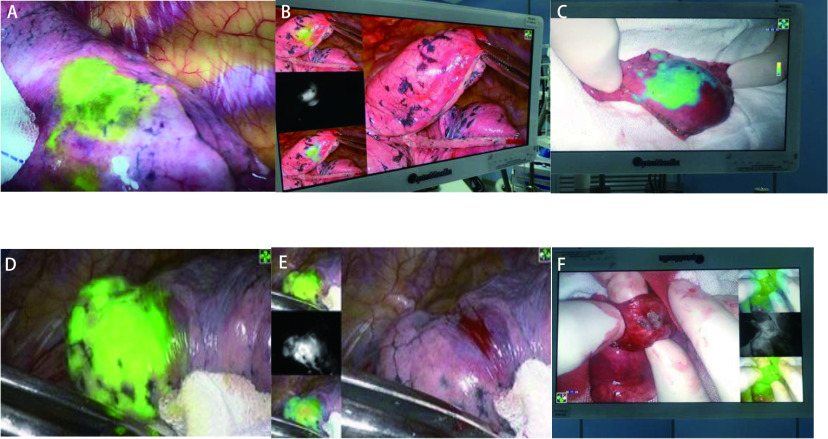
荧光腔镜下肺结节视图。A、D：进胸腔后荧光腔镜下肺结节呈荧光绿染; B、E:荧光腔镜3种模式视图下肺结节呈现图；C、F：肺结节行楔形切除术后，在荧光下呈绿色，切开可明显见肺异常组织 Lung nodules under fluorescent endoscope. A and D: pulmonary nodules showed fluorescent green staining under fluorescent endoscopy after admission into the chest cavity. B and E: pulmonary nodules were shown in the three modes of fluorescent endoscopy. C and F: after wedge resection of pulmonary nodules, the lung tissue where the nodules were located was green under fluorescence, and abnormal lung tissues could be clearly seen after incision.

### 1.2 数据统计与分析

统计患者的人口学特征、病史、家族史、结节特征、手术方式和病理诊断等信息，连续性变量的均值使用（Mean±SD）表示，分类变量使用频率表示。使用SPSS 20.0统计软件进行统计分析。

## 2 结果

### 2.1 患者情况

2018年12月-2019年8月共16例入组，其中男性7例，女性9例，平均年龄（56.12±12.74）岁，既往有肿瘤病史5例，既往有家族史10例，吸烟史4例，就诊伴有症状者5例，就诊肿瘤标志物检查升高者6例，CT检查肺上只有1处病灶的患者最多，有9例，有多处病灶的患者有7例，其中2处病灶2例，3处病灶患者3例，4处病灶患者2例（表 1）。结节特点：16例患者胸部CT显示30处病灶，结节最大径的平均值为（11.12±3.65）mm，其中位于右上肺的结节最多，有13个结节，左下肺最少，有3个结节，纯磨玻璃结节有20个，部分实性结节8个，实性结节2个。结节距离肺膜表面的平均距离为（15.72±8.01）mm（表 2）。

**2:** 肺部结节特点（*n*=30） Characteristic of pulmonary lesions (*n*=30)

Variables	Data
Location	
RUL	13 (43.3%)
RML	3 (10.0%)
RLL	6 (20.0%)
LUL	5 (16.7%)
LLL	3 (10.0%)
Composition	
GGO	20 (66.7%)
Part-solid	8(26.7%)
Solid	2(6.6%)
Maximum diameter	(11.12±3.65)min
Distance to pleura surface	(15.72±8.01)min
The average interval time	(25.00±5.29)min
The average location time	(12.06±2.74)min
RUL: right upper lung; RML: right middle lung; RLL; right lower lung; LUL: left upper lung; LLL: left lower lung.	

### 2.2 手术相关结果

2018年12月-2019年8月期间，采用ENB引导下注入吲哚箐绿定位肺内小结节患者16例，考虑到部分多发肺结节患者病灶位于双肺，同时切除患者风险及术后并发症较大，另有患者因手术风险拒绝行双肺部结节同时切除，16例患者共定位20例结节，平均定位时间（12.06±2.74）min，定位成功率100.00%，在荧光腔镜下探查发现荧光都位于离肿物最近的肺膜表面，术中发现染料播散胸腔1例，无定位后肺出血等并发症。所有患者均先在荧光腔镜下将呈现荧光的肺组织切除，送冰冻检查，根据冰冻结果选择手术方式，行肺叶切除患者9例，亚肺叶切除患者7例（其中包含1例患者冰冻诊断为浸润性腺癌，既往行左下肺切除病史，患者术前肺功能难以耐受肺叶切除），术后标本经有经验的病理科医生确认，诊断为肿瘤病变患者15例，其中浸润性腺癌12例，原位腺癌5例，类癌1例，炎症2例，所有的病例的最终诊断跟术中冰冻切片结果一致。其中8处结节行肺叶切除，12处行楔形切除，1处行肺段切除（表 3）。

**3:** 最终病理结果及手术方式（*n*=20） Final pathologic evaluation and operation procedure (*n*=20)

Variables	Data
Pathologic diagnosis	
Inflammatory	2 (10.0%)
Adenocarcinoma in situ	5 (25.0%)
Invasive adenocarcinoma	12 (60.0%)
Carcinoid	1 (5.0%)
Operation procedure	
Lobectomy	8 (40.0%)
Wedge resection	11 (55.0%)
Segmental resection	1 (5.0%)

## 3 讨论

随着低剂量螺旋CT应用于肺癌的早期筛查，诊断早期肺癌患者越来越多，胸腔镜下肺叶或亚肺叶切除已经成为肺小结节的常规手术，大部分肺内结节因其体积小，无实性成分，跟正常肺组织区别度低，手术过程中外科医生难以触摸，术中通过触诊定位失败为63%^[10]^，常常因定位失败不得已而转开胸手术，或直接行患者肺叶切除，这显然增加了患者的创伤，损失了患者的肺功能，既往研究表明在部分纯GGO患者中使用亚肺叶切除的长期生存率不低于肺叶切除^[11]^。因此为了解决这个问题，国际上各种肺内小结节术前定位方法应运而生。

目前较为常用的术前定位方法包括：Hook-wire（CT引导下经皮肤穿刺钩定位法）、CT引导下注射核素、染料、放置弹簧圈以及术中借助超声装置等。也有报道通过ENB引导注入亚甲兰、生物胶及靛卡红等染料进行定位^[8, 12-14]^，此外也有研究将亚甲蓝和生物蛋白胶混合注入结节部位，可在肺表面形成可触及的蓝染区域^[15]^，但可能会影响病理科医生寻找肿物且部分患者可能会对生物蛋白胶有潜在的过敏反应，各有优缺点。在这项研究中，通过ENB引导注入荧光染料定位直径 < 20mm的肺结节，然后立马行腔镜下手术切除，靶病灶染色成功率100%，所有定位结节均成功切除，除1例患者术中因麻醉原因出现咳嗽导致引导管刺穿肺组织出现染料扩散，在荧光腔镜下整个肺表面出现大面积荧光绿染，难以区分结节位置，不利于实行肺段切除，但不影响术中楔形切除明确病理，因为在荧光腔镜普通视图下，可以观察到肺膜表面刺穿的出血点，之前提到将ICG原液注入距结节0.5cm处，外科医生可将刺穿处周围肺组织楔形切除，也可将结节顺利切除明确病理，此外尽量控制ICG注入剂量可能对一旦刺穿肺染料进入胸腔减少播散面积有帮助，我们中心经验一般将ICG原液0.1mL注入肺结节周围能达到最好手术效果。所以术中充分麻醉，以及有经验的内镜医生操作很有必要，特别是对术前打算行肺段切除的患者。荧光染料不同于既往美兰与蛋白胶混合类染色剂，前者注入肺组织后不会影响肺结节及周围正常的肺组织结构，只有在荧光下呈现绿色染色，自然状态下不会给病理科医生染色及诊断带来不必要的麻烦，且术后外科医生会再次在荧光下再次识别标记结节，病理科医生可直接按标记位置取材。此外在手术过程中定位导航时间及等待手术切除间隔时间未显著延长总手术时间，在正常手术室就可以操作，不增加运营成本。

本研究我们可以总结得到以下经验：①ENB引导下定位操作需在充分麻醉下及富有经验的内镜医生下进行；②ENB引导下注入荧光染料定位可以更加精准定位，且染料不会影响病理诊断；③术中使用荧光染料应减少荧光剂的剂量，可以较少染料进入胸腔后的播散。

综上所述，ENB引导下荧光染色定位技术是一种损伤小、定位精准、并发症低的一项新技术，能够提高目前微创外科下的精准切除率，临床上部分患者因术前心肺功能难以耐受肺叶切除，解剖性肺段切除成为这部分患者的首选。结合目前行解剖性肺段切除时，如何判断肿瘤所在肺段位置，如何区分肺段之间分界，做到肿瘤根治同时不增加患者术后出现漏气等并发症的风险这些热点问题，ENB的精准定位并结合患者术前3D重建结果，有望成为解剖性亚肺叶切除定位的首选方法。相信通过技术经验的积累及后续病例的不断扩充，此项技术将获得广泛推广。
